# Neurophysiological Characteristics of Nitrous Oxide‐Induced Polyneuropathy: A Case Series

**DOI:** 10.1111/jns.70156

**Published:** 2026-07-31

**Authors:** Tanya Elisabeth Bentley, Volker Limmroth, Maximilian Zimmermann, Johannes Fabian Holle

**Affiliations:** ^1^ Department of Neurology Hospitals of the City of Cologne Cologne Germany; ^2^ Department of Psychiatry University of Cologne, Medical Faculty Cologne Germany; ^3^ Health Faculty/Department for Human Medicine University of Witten/Herdecke Witten Germany; ^4^ Lung Clinic Hospitals of the City of Cologne Cologne Germany

**Keywords:** electrodiagnostic studies, methylmalonic acid, nitrous oxide, polyneuropathy, vitamin B12

## Abstract

**Background:**

Nitrous oxide (N_2_O) misuse is a growing health concern, with N_2_O‐induced neurological disorders increasingly reported across Europe. Among these, N_2_O‐induced polyneuropathy (PNP) can lead to permanent deficits, yet its neurophysiological characteristics remain inconsistently described. This retrospective case series aimed to characterize the electrophysiological pattern of N_2_O‐induced polyneuropathy.

**Methods:**

We reviewed the records of 12 patients with acute neurological symptoms and a history of N_2_O abuse, all of whom underwent electrophysiological testing at a German tertiary care hospital between July 2023 and December 2024. Data were analyzed descriptively.

**Results:**

Conduction studies revealed evidence of PNP in eight out of 12 patients and the most common pattern was pure motor (5/8). No distinct clinical phenotype could be identified for patients with electrophysiologically confirmed PNP or Pure‐Motor PNP. SSEPs were abnormal in all assessable patients, whereas signs of SCD on MRI were present in a smaller proportion. Only three of 10 tested patients had reduced vitamin B12 levels, while all showed elevated methylmalonic acid (MMA) levels. Mean MMA levels were higher in patients with PNP than in those without.

**Interpretation:**

The reason why N_2_O leads to different patterns of PNP remains unclear and a distinct phenotype for N_2_O‐induced PNP or Pure‐Motor PNP was not found in this cohort. MMA represents a more reliable biomarker than serum vitamin B12 in the diagnostic workup of N_2_O‐induced neurotoxicity. These findings highlight the need for further research into additional neurotoxic properties of N_2_O and individual predisposing factors underlying the different electrophysiological patterns of N_2_O‐induced PNP.

## 1 Introduction

The recreational abuse of nitrous oxide (N_2_O), commonly known as “laughing gas,” has increased in recent years [1]. According to the 2023 Crime Survey for England and Wales, N_2_O was the third most prevalent drug among young adults aged 16–24, following cannabis and powder cocaine, with a prevalence of 4.2% [2]. Until recently, N_2_O was legally, largely unregulated and inexpensively available in Germany, sold in balloons, bulbs, or canisters, which contributed to its popularity, especially among adolescents and young adults [1]. A survey conducted in Frankfurt am Main, a large German city, revealed a significant increase in the lifetime prevalence of N_2_O abuse among adolescents aged 15–18 years, from 7% in 2020 to 17% in 2022 [3]. In response to this rising misuse, an amendment to the German New Psychoactive Substances Act came into effect in April 2026, prohibiting the supply of N_2_O to minors and its distribution via vending machines and mail order. Adults may still purchase a limited number of small capsules containing up to 8.4 g each [4]. N_2_O abuse is also increasingly reported outside of Europe and can be considered an emerging global health concern [5, 6]. N_2_O is a colorless, nonirritating gas with relaxing effects as well as hallucinogenic and euphoriant properties [1, 7]. It is used in clinical practice as an inhaled anesthetic and analgesic, which induces its physiological effects within seconds of inhalation [6, 8]. The effects typically last minutes, allowing users to resume normal function within a short amount of time [9]. This can lead to users repeating the inhalation several times without realizing the total intake and the increased likelihood of adverse effects [10]. Long‐term abuse can lead to severe neurological sequelae, including nervous system damage such as subacute combined degeneration of the spinal cord (SCD) and polyneuropathy (PNP), psychiatric disorders, hematological abnormalities as well as thromboembolic complications [6, 11, 12]. A drastic increase of N_2_O‐induced neurological disorders was reported in the greater Paris area in 2022, exceeding the incidence of myelopathies of other causes or Guillain‐Barré syndrome (GBS) [13]. Although N_2_O‐induced PNP has most commonly been described as sensorimotor, reported patterns vary across the current literature [14, 15, 16, 17, 18, 19, 20, 21].

In the following, we report a retrospective case series of 12 patients who presented with acute neurological symptoms and a history of N_2_O abuse. The aim of this case series was to analyze the neurophysiological characteristics of N_2_O‐induced PNP.

## 2 Materials and Methods

### 2.1 Data Collection, Inclusion and Exclusion Criteria and Endpoints

This monocentric case series was conducted retrospectively. Patients were identified through a systematic search of the electrophysiological laboratory database. All patients who underwent electrophysiological testing between 1 July 2023 and 31 December 2024 at the Department of Neurology of a German tertiary care hospital were screened.

The inclusion criteria were as follows: age 18 years or above, history of N_2_O abuse over a minimum of 3 weeks in the preceding 6 months, new onset of neurological symptoms temporally associated with N_2_O consumption, and completion of nerve conduction studies. As the aim of this case series was to analyze the electrophysiological pattern of N_2_O‐induced PNP, patients were excluded if they underwent electrophysiological testing, but nerve conduction studies (NCS) were incomplete.

The primary endpoint was the presence and pattern of PNP in NCS. The population was divided into two groups according to the results of NCS: Patients who showed signs of PNP and patients who did not show signs of PNP. The secondary endpoint was to assess differences between the two groups concerning clinical presentation, the presence of SCD on MRI, somatosensory evoked potentials (SSEP) findings and biomarkers.

### 2.2 Diagnostic Assessment

Concerning the primary endpoint, NCS were performed on the tibial motor nerves, the sural sensory nerves, either the left or right median motor and sensory nerve. The tibial and sural nerves were selected according to the protocol of our electrophysiological laboratory and were preferred over the fibular nerve for motor NCS and over the superficial fibular nerve for sensory NCS, as both fibular nerves are more susceptible to entrapment neuropathy. Measurements were compared to the conduction study reference values of the local electrophysiological laboratory as shown in Table 1. PNP was defined by abnormal nerve conduction findings or abnormal F‐wave responses in at least one nerve tested bilaterally. F‐wave responses were assessed using the F‐M latency (defined as the minimal F‐wave latency minus the distal motor latency), compared to height‐dependent reference values used in our laboratory. F‐wave responses were considered abnormal if the F‐M latency exceeded the reference values or if no F‐wave could be elicited. While isolated F‐wave abnormalities are not part of the standard electrodiagnostic criteria for PNP, we included them as an indicator of early PNP given the young cohort without relevant prior neurological conditions. Borderline values of NCS and F‐M‐latencies were interpreted in the context of the overall electrophysiological findings rather than as isolated criteria for PNP classification. In the case of PNP, an examination of the exact pattern was conducted, investigating length‐dependence, axonal, demyelinating or axonal‐demyelinating pattern and sensory, motor or sensorimotor pattern. Length‐dependence was defined as the presence of abnormal nerve conduction findings in the distal lower limbs (LL) in the absence of upper limb (UL) involvement. To determine whether a PNP had a demyelinating or axonal pattern, the modified criteria for motor conduction studies for demyelination as outlined in the German neurological guideline, adapted from van den Bergh et al., were employed [22, 23]. Pure‐Motor PNP was defined as pathological motor conduction studies with normal sensory conduction studies. F‐M‐latency abnormalities were considered supportive but not sufficient as the sole criterion for this classification. Bilateral amplitude‐reduction of the tibial nerve was accepted as sufficient evidence of motor PNP given the young age of our cohort and the low likelihood of alternative explanations, such as cauda equina compression due to lumbar spinal stenosis. Motor‐dominant PNP was defined as more pronounced pathological motor conduction studies and of the tibial nerves compared with only mildly pathological sensory conduction studies of the sural nerves, while sensory‐dominant PNP was defined as the inverse pattern.

**TABLE 1:** Nerve conduction study results for each patient.

Parameter	Ref.	Pat. 1	Pat. 2	Pat. 3	Pat. 4	Pat. 5	Pat. 6	Pat. 7	Pat. 8	Pat. 9	Pat. 10	Pat. 11	Pat. 12
Tib. amp. L [mV]	≥ 6	11.0	11.5	**0**	**4.7**	16.7	7.6	**5.5**	14.1	**3.5**	9.6	**0.14**	**5.1**
Tib. NCV L [m/s]	≥ 39	44	44	**35**	40	40	42	**38**	42	**38**	40	**31**	40
Tib. F‐M latency L		**↑**	n	**—**	n	n	**↑**	n	n	n	n	**—**	n
Tib. amp. R [mV]	≥ 6	10.0	13.5	**0.1**	**4.7**	19.0	7.1	**5.1**	10.5	**1.2**	9.8	**0.1**	**5.8**
Tib. NCV R [m/s]	≥ 39	42	46	**36**	40	**38**	**38**	**37**	41	41	**38**	**36**	41
Tib. F‐M latency R		**↑**	n	**—**	n	n	**↑**	**↑**	n	n	n	**—**	n
Sural amp. L [μV]	≥ 5	**2.4**	20.8	12.5	12.5	12.7	**4.9**	14.7	4.3	5.1	11.1	**4.5**	10
Sural NCV L [m/s]	≥ 43	**40**	43	46	48	45	**40**	46	50	**42**	43	**31**	54
Sural amp. R [μV]	≥ 5	**2.4**	9.3	26.8	13.2	12.5	11.2	16.8	7.2	10.6	21.4	**1.4**	10
Sural NCV R [m/s]	≥ 43	**39**	44	44	47	41	43	44	47	42	45	**34**	52
Med. mot. amp. L/R [mV]	≥ 5	6.9	8.5	7.7	8.1	10.1	10.3	15.4	9.7	16.7	7.1	19.4	22.1
Med. mot. NCV L/R [m/s]	≥ 47	48	57	48	50	52	50	50	56	50	54	49	59
Med. sens. amp. L/R [μV]	≥ 8	18.1	28.7	28.3	24.6	23.6	12.2	23.2	14.2	36.3	**7.1**	19.5	24.6
Med. sens. NCV L/R [m/s]	≥ 44	55	49	57	51	59	46	57	50	57	54	50	55
PNP pattern		**Sensorimotor (sensory‐dominant) axonal**	No PNP	**Pure motor axonal**	**Pure motor axonal**	No PNP	**Early stage**	**Pure motor axonal**	No PNP	**Pure motor axonal**	No PNP	**Sensorimotor (motor‐dominant) axonal**	**Pure motor axonal**

*Note:* Bold values indicate deviation from reference range.

Abbreviations: — = absent (no F‐wave elicited), ↑ = prolonged, amp. = amplitude, F‐M = F‐minus‐M‐wave, L = left, m/s = meters per second, Med. mot. = median motor nerve, Med. sens. = median sensory nerve, mV = millivolt, n = normal, NCV = nerve conduction velocity, R = right, Tib. = tibial nerve, μV = microvolt.

Concerning the secondary endpoints, presenting symptoms as documented in the medical records were assessed, including whether the patient reported sensory symptoms, motor weakness or gait disturbance. Additionally, findings of the neurological examination were assessed. Due to the heterogeneous level of detail in the documentation of neurological examination findings across medical records, sensory deficits of the upper and lower extremities were summarized as any sensory deficit present, regardless of modality (hypaesthesia, paraesthesia, or pallhypaesthesia). Similarly, gait disturbance was recorded as present if any abnormality was noted across the components of gait examination, including regular gait, tandem gait, heel walk, and toe walk. Neuroimaging was used to assess evidence of SCD. Signs of SCD were defined as symmetric hypointensity on T1‐weighted images and hyperintensity on T2‐weighted images, involving the posterior columns of the spine, showing the inverted V sign on axial MRI [17]. SSEPs of the lower extremities were used to assess involvement of the somatosensory pathway. Latencies were obtained by stimulation of the posterior tibial nerve or the sural nerve, with recording of the P40 response from the contralateral somatosensory cortex using needle electrodes. The recorded latency was adjusted to body height using the formula: P40 latency/height in meters (reference: < 25 ms). SSEPs were classified as pathological if no reproducible cortical response could be obtained or if the height‐adjusted P40 latency exceeded the reference value. As SSEPs reflect the integrity of the entire somatosensory pathway, abnormal SSEP findings were considered indicative of somatosensory pathway involvement but did not allow a distinction between SCD and PNP.

Assessed laboratory markers included vitamin B12, methylmalonic acid (MMA), hemoglobin, Mean Corpuscular Volume (MCV) and Mean Corpuscular Hemoglobin (MCH). For patients with vitamin B12 levels below the detection limit of the assay (reported as < 148 pg/mL), a value of 148 pg/mL was used for the calculation of mean vitamin B12 levels. Macrocytic hyperchromic anemia was defined as reduced hemoglobin in combination with elevated MCV and elevated MCH.

### 2.3 Analysis

Given the small sample size, data were analyzed descriptively without formal statistical testing. Descriptive analysis of numerical values including calculation of mean values and standard deviation were performed in Microsoft Excel 2024 Microsoft Corporation. Tables were created in Microsoft Word 2024 Microsoft Corporation and Microsoft Excel 2024 Microsoft Corporation.

## 3 Results

### 3.1 Demographic Data and Primary Endpoint: PNP

The inclusion criteria concerning age and history were met by 13 patients. One patient was excluded from the case series because of discharge prior to the completion of NCS. Twelve patients were included in this case series. The mean age was 22 years (±3.3), and 11 of the 12 patients were male.

Table 1 presents the results of NCS and the diagnosed PNP patterns. Of the 12 patients, eight (67%) met the neurophysiological criteria for PNP, while four (33%) showed no evidence of PNP. Among the eight patients with PNP, five (62.5%) demonstrated a length‐dependent purely motor axonal pattern, one (12.5%) showed an early stage PNP, one (12.5%) presented with a length‐dependent sensorimotor axonal pattern with sensory dominance, and one (12.5%) with a length‐dependent sensorimotor axonal pattern with motor dominance.

### 3.2 Secondary Endpoints: Clinical Presentation, MRI, SSEP and Biomarkers

Table 2 presents the clinical, neuroimaging, SSEP, and laboratory findings. Based on the primary endpoint, the cohort was divided into PNP (*n* = 8) and Non‐PNP (*n* = 4) groups. Given the predominance of a pure motor axonal pattern among PNP patients (5/8, 62.5%), a further analysis was performed comparing Pure‐Motor PNP (*n* = 5) with Non‐Pure‐Motor PNP (*n* = 3).

**TABLE 2:** Clinical, MRI, SSEP and laboratory findings by group.

Parameter	All (*n* = 12)	Non‐PNP (*n* = 4)	PNP (*n* = 8)	Pure‐potor PNP (*n* = 5)	Non‐pure‐motor PNP (*n* = 3)
Reported symptoms					
Sensory symptoms	12/12 (100%)	4/4 (100%)	8/8 (100%)	5/5 (100%)	3/3 (100%)
Motor weakness	10/12 (83%)	4/4 (100%)	6/8 (75%)	4/5 (80%)	2/3 (67%)
Gait disturbance	10/12 (83%)	3/4 (75%)	7/8 (88%)	4/5 (80%)	3/3 (100%)
Neurological examination					
Cranial nerve deficit	0/12 (0%)	0/4 (0%)	0/8 (0%)	0/5 (0%)	0/3 (0%)
Any sensory deficit UL	5/11 (45%)^a^	2/3 (67%)^a^	3/8 (38%)	2/5 (40%)	1/3 (33%)
Any sensory deficit LL	11/12 (92%)^a^	4/4 (100%)	7/8 (88%)^a^	5/5 (100%)	2/3 (67%)^a^
UL motor deficit	1/12 (8%)	0/4 (0%)	1/8 (12%)	1/5 (20%)	0/3 (0%)
LL motor deficit	5/12 (42%)	1/4 (25%)	4/8 (50%)	3/5 (60%)	1/3 (33%)
UL absent/reduced reflexes	2/11 (18%)^a^	0/4 (0%)	2/7 (29%)^a^	1/5 (20%)	1/2 (50%)^a^
LL absent/reduced reflexes	4/12 (33%)	2/4 (50%)	2/8 (25%)	1/5 (20%)	1/3 (33%)
Reflex level discrepancy (LL vs. UL)	6/11 (55%)^a^	3/4 (75%)	3/7 (43%)^a^	1/5 (20%)	2/2 (100%)^a^
Babinski sign	0/12 (0%)	0/4 (0%)	0/8 (0%)	0/5 (0%)	0/3 (0%)
Gait disturbance	10/12 (83%)	3/4 (75%)	7/8 (88%)	5/5 (100%)	2/3 (67%)
Romberg's test pathological	8/11 (73%)^a^	2/4 (50%)	6/7 (86%)^a^	4/4 (100%)^a^	2/3 (67%)
MRI					
SCD on cervical MRI	8/11 (73%)^a^	1/3 (33%)^a^	7/8 (88%)	5/5 (100%)	2/3 (67%)
Somatosensory evoked potentials (SSEPs)					
SSEP pathological	11/11 (100%)^a^	3/3 (100%)^a^	8/8 (100%)	5/5 (100%)	3/3 (100%)
Biomarkers					
Vitamin B12 [pg/mL; ref. 187–883]	251.5 ± 120.5^a^	261.0 ± 54.9	245.2 ± 155.5^a^	283.0 ± 186.0^a^	169.5 ± 2.1^a^
Vitamin B12 reduced	3/10 (30%)^a^	0/4 (0%)	3/6 (50%)^a^	1/4 (25%)^a^	2/2 (100%)^a^
MMA [μg/L; ref. 9–32]	429.4 ± 279.9^a^	395.4 ± 204.9	452.2 ± 338.0^a^	314.3 ± 316.1^a^	727.8 ± 208.5^a^
MMA elevated	10/10 (100%)^a^	4/4 (100%)	6/6 (100%)^a^	4/4 (100%)^a^	2/2 (100%)^a^

Abbreviations: UL = upper limb; LL = lower limb; SCD = subacute combined degeneration; PNP = polyneuropathy; MMA = methylmalonic acid; pg/mL = picograms per milliliter; μg/L = micrograms per liter; ref. = reference range.

^a^ Not assessable in all patients due to incomplete documentation.

All 12 patients reported sensory symptoms upon presentation. On neurological examination, sensory deficits of the LL were found in 11/12 patients (92%), and sensory deficits of the UL in 5/11 patients (45%). No patient presented with UL sensory deficits in the absence of LL involvement. No substantial differences in sensory findings were observed between the PNP and Non‐PNP group, or between Pure‐Motor PNP and Non‐Pure‐Motor PNP group. Motor weakness upon presentation was reported by 10/12 patients (83%) overall. On neurological examination, motor deficits were found in 5/12 patients overall (42%), including 4/8 PNP patients (50%) and 1/4 Non‐PNP patients (25%), with the Pure‐Motor PNP group showing motor deficits in 3/5 (60%) compared to 1/3 in the Non‐Pure‐Motor PNP group (33%). Gait disturbance was reported by 10/12 patients overall (83%). On neurological examination, gait disturbance was found in 10/12 patients (83%), with comparable rates across groups. Romberg's test was pathological in 8/11 patients (73%) overall, more frequently in the PNP group (6/7, 86%) than in the Non‐PNP group (2/4, 50%). Absent or reduced reflexes were found in 4/12 patients overall (33%), with comparable rates across groups. A reflex level discrepancy between lower and upper extremities was observed in 6/11 assessed patients (55%), without substantial differences between the PNP and Non‐PNP groups. No patient showed a positive Babinski sign. No patient showed cranial nerve involvement.

SCD on cervical MRI was found in 8/11 assessed patients overall (73%). SCD on MRI was more frequent in the PNP group (7/8, 88%) compared to the Non‐PNP group (1/3, 33%). Within the PNP subgroups, all five Pure‐Motor PNP patients showed evidence of SCD (100%), compared to 2/3 Non‐Pure‐Motor PNP patients (67%). SSEP of the lower extremities were pathological in all 11 assessed patients (100%).

Biomarkers were available in 10/12 patients prior to vitamin B12 substitution. MMA was elevated in all 10 patients (100%). Mean MMA levels (reference: 9–32 μg/L) were 395.4 ± 204.9 μg/L in the Non‐PNP group and 452.2 ± 338.0 μg/L in the PNP group, with the Non‐Pure‐Motor PNP group showing the highest mean levels (727.8 ± 208.5 μg/L) compared to the Pure‐Motor PNP group (314.3 ± 316.1 μg/L). Vitamin B12 was reduced in 3/10 patients (30%) overall, with no reductions observed in the Non‐PNP group (0/4) compared to 3/6 PNP patients (50%).

Hemoglobin was reduced in one patient of the Non‐PNP group, but no patients met the criteria for macrocytic anemia.

## 4 Discussion

Regarding the primary endpoint, 67% of our patients showed electrophysiological evidence of PNP. In comparison with the literature, however, the proportion of patients classified as having electrophysiological PNP depends strongly on the diagnostic criteria applied. Hassing et al., applying more precise criteria (reduced amplitudes in ≥ 2 nerves), reported electrodiagnostic abnormalities in up to 91% of patients, with 74% fulfilling their formal PNP criteria [24]. As we considered consistent bilateral abnormalities in a single nerve sufficient for an electrophysiological diagnosis of PNP, some of our cases might not have met PNP criteria under these criteria. Notably, 62.5% of the PNP group displayed a pure motor pattern. Previous studies have described heterogeneous electrophysiological phenotypes of N_2_O‐induced PNP, including sensory, sensorimotor, and pure motor forms [14, 15, 16, 17, 18, 19, 20, 21, 22, 24, 25, 26]. While sensorimotor neuropathy is most reported, several cohorts have also demonstrated predominantly or exclusively motor involvement [26, 27]. Taken together, the available literature suggests that N_2_O‐induced PNP does not represent a single electrophysiological entity but rather comprises different phenotypic patterns. This variability is difficult to reconcile with the classical understanding of vitamin B12 deficiency–associated neuropathy, which is typically sensory‐predominant [27, 28]. Since N_2_O causes a functional vitamin B12 deficiency through irreversible inactivation of the vitamin, accumulation of MMA and homocysteine may impair myelin synthesis and lead to axonal injury [28, 29, 30, 31, 32]. However, this mechanism alone does not adequately explain the occurrence of pure motor neuropathy. Importantly, the reason why some patients develop sensory or sensorimotor neuropathy whereas others show a pure motor pattern remains unclear. A direct toxic effect of N_2_O in combination with various predisposing factors, such as genetic polymorphisms, is a possibility [33, 34, 35]. Furthermore, Hassing et al. proposed that the combination of prominent sensory symptoms and selective motor abnormalities might indicate involvement of the nerve roots [24]. However, given the absence of a routine electromyographic examination of the paravertebral muscles, it is not possible to draw definitive conclusions regarding nerve root involvement in our cohort.

The analysis of the secondary endpoints revealed no meaningful differences in clinical presentation between the patients with and without electrophysiologically confirmed PNP. Thus, the clinical phenotype alone does not allow reliable prediction of the presence or absence of PNP in N_2_O‐associated neurotoxicity. Regarding differences in MRI findings, signs of SCD were more frequently observed in the PNP group. However, no clear association between specific PNP patterns and the occurrence of SCD could be established. In our cohort, SSEPs of the lower extremities were pathological in all assessable patients, including all patients in the Non‐PNP group, in whom NCS showed no evidence of PNP and spinal MRI was unremarkable in the majority. This dissociation may indicate that SSEPs are a highly sensitive marker of N_2_O‐induced neurological involvement and may detect impairment of the somatosensory pathway before it becomes apparent as PNP on NCS or as SCD on MRI. However, this sensitivity comes at the cost of specificity. As SSEPs reflect the integrity of the entire somatosensory pathway, an abnormal result cannot localize the underlying lesion and does not distinguish PNP from SCD. While assessment of the spinal N22 potential could aid this distinction, it was not assessable in most of our patients, so that it was not included in our analysis. Moreover, abnormal SSEPs are not specific to N_2_O‐induced sequelae and may result from any lesion along the somatosensory pathway, including central causes unrelated to N_2_O. As cranial MRI was not routinely available in our cohort, such alternative central causes could not be formally excluded. SSEPs are therefore of limited value as a standalone marker and require correlation with NCS and MRI. Within these limitations, abnormal SSEPs in the absence of PNP or SCD may indicate early N_2_O‐induced involvement of the somatosensory system that is not yet detectable by standard electrophysiological or imaging studies. MMA levels appeared higher in PNP patients than in Non‐PNP patients, consistent with previous reports linking elevated MMA levels to greater disease severity [36]. This finding raises the possibility that the development of PNP in N_2_O‐associated neurotoxicity may be associated with a higher degree of metabolic disturbance. Although mean MMA levels were numerically higher in the Non‐Pure‐Motor than in the Pure‐Motor PNP group, only two patients in the Non‐Pure‐Motor group had available MMA values, which precluded any statistical comparison between PNP subtypes, the underlying mechanisms responsible for the different electrophysiological patterns thus remain unknown. Vitamin B12 serum levels and hematological markers were of limited diagnostic value in our cohort. Reduced vitamin B12 levels were observed in only a minority of patients, and none showed macrocytic hyperchromic anemia, consistent with previous reports [21, 37, 38, 39].

These findings support the concept that N_2_O primarily causes functional rather than absolute vitamin B12 deficiency. Consequently, MMA appears to be a more sensitive biomarker in this setting.

It is advisable to routinely screen for N_2_O abuse in young adults presenting with acute neurological symptoms compatible with PNP or myelopathy. Despite vitamin B12 supplementation, permanent neurological deficits may result from N_2_O abuse [24, 40]. Given the rising prevalence of N_2_O misuse, awareness among healthcare professionals and the public is essential to promote early recognition and intervention [24, 40].

This study has several limitations. The retrospective design and small sample size limit the generalizability of our findings. Moreover, data on the total number of patients presenting with N_2_O abuse were not assessed and therefore the proportion of patients who underwent electrophysiological testing could not be determined. In addition, incomplete documentation of neurological findings and inconsistent data regarding duration and amount of N_2_O exposure precluded analysis of dose–response relationships.

### 4.1 Conclusion

The most common electrophysiologically evident pattern of N_2_O‐induced PNP was a length‐dependent pure motor axonal pattern in our cohort. While patients with PNP showed higher MMA levels, more frequently reduced vitamin B12 levels, and more frequent signs of SCD on MRI compared to Non‐PNP patients, no distinct clinical phenotype could be identified for N_2_O‐induced PNP.

The variability in electrophysiological presentation of N_2_O‐induced PNP, and the high proportion of Pure‐Motor PNP observed in our cohort, may itself serve as a starting point for investigating additional neurotoxic properties of N_2_O and exploring individual predisposing factors. Larger prospective studies are needed to further elucidate these mechanisms.

## Author Contributions

T.E.B. conceptualized the case series, curated the clinical data, performed the formal analysis and investigation, and drafted the original manuscript. V.L. and M.Z. contributed to the conceptualization, provided supervision, and critically reviewed the manuscript. J.F.H. conceptualized the case series, supervised and administered the project, validated the results, and critically reviewed and edited the manuscript. All authors read and approved the final manuscript.

## Funding

The authors have nothing to report.

## Ethics Statement

This human study was performed in accordance with the Declaration of Helsinki and approved by the Ethics Committee of the University Witten/Herdecke (application number: S‐287/2024, approval: 18.11.2024). Patient consent was waived by the Ethics Committee due to the retrospective nature of this case series, as only existing patient data were analyzed without any intervention or additional patient contact.

## Conflicts of Interest

The authors declare no conflicts of interest.

## Data Availability

The data that support the findings of this study are available on request from the corresponding author. The data are not publicly available due to privacy or ethical restrictions.
